# Fast PET reconstruction with variance reduction and prior-aware preconditioning

**DOI:** 10.3389/fnume.2025.1641215

**Published:** 2025-09-17

**Authors:** Matthias J. Ehrhardt, Zeljko Kereta, Georg Schramm

**Affiliations:** ^1^ Department of Mathematical Sciences, University of Bath, Bath, United Kingdom; ^2^ Computer Science Department, University College London, London, United Kingdom; ^3^ Department of Imaging and Pathology, KU Leuven, Leuven, Belgium

**Keywords:** PET, MAP, preconditioning, variance reduction, stochastic gradient methods, regularization methods, image reconstruction

## Abstract

We investigated subset-based optimization methods for positron emission tomography (PET) image reconstruction incorporating a regularizing prior. PET reconstruction methods that use a prior, such as the relative difference prior (RDP), are of particular relevance because they are widely used in clinical practice and have been shown to outperform conventional early-stopped and post-smoothed ordered subset expectation maximization. Our study evaluated these methods using both simulated data and real brain PET scans from the 2024 PET Rapid Image Reconstruction Challenge (PETRIC), where the main objective was to achieve RDP-regularized reconstructions as fast as possible, making it an ideal benchmark. Our key finding is that incorporating the effect of the prior into the preconditioner is crucial for ensuring fast and stable convergence. In extensive simulation experiments, we compared several stochastic algorithms—including stochastic gradient descent (SGD), stochastic averaged gradient amelioré (SAGA), and stochastic variance reduced gradient (SVRG)—under various algorithmic design choices and evaluated their performance for varying count levels and regularization strengths. The results showed that SVRG and SAGA outperformed SGD, with SVRG demonstrating a slight overall advantage. The insights gained from these simulations directly contributed to the design of our submitted algorithms, which formed the basis of the winning contribution to the PETRIC 2024 challenge.

## 1 Introduction

### 1.1 Context

Positron emission tomography (PET) is a pillar of modern clinical imaging, widely used in oncology, neurology, and cardiology. Most state-of-the-art approaches for the image reconstruction problem in PET imaging can be cast as an optimization problem

(1) $$x^{*}\in \underset{x}{\arg\;\min}\;\;\left\{D(Ax+r,y)+R(x)\right\},$$

where the data fidelity term D:Y×Y→[0,∞] measures how well the estimated data Ax+r matches the acquired data y and the regularizer R:X→[0,∞] penalizes unwanted features in the image. A:X→Y is a linear forward model for the PET physics, which includes effects such as scanner sensitivities or attenuation, and r is the additive background term to account for scattered and random coincidences. Due to the Poisson nature of the data, the data fidelity is usually taken as the Kullback–Leibler (KL) divergence. The regularizer commonly entails non-negativity constrains and terms that promote smoothness. A particularly successful model for smoothness in PET is the relative difference prior (RDP) (1).

This paper focuses on algorithms for the fast reconstruction of $x^{*}$. Particularly, we present our winning contribution to the 2024 PET Rapid Image Reconstruction Challenge (PETRIC) (2), where the task was to reconstruct data from various PET scanners using RDP-regularized reconstruction methods. PET image reconstructions that use the RDP are of particular current relevance because RDP is widely used in clinical practice, being implemented by a major commercial vendor, and has been shown to outperform conventional early-stopped and post-smoothed ordered subset-maximum likelihood expectation maximization (OS-MLEM) reconstructions (3–5). Although implementations based on block sequential regularized expectation maximization (BSREM) (6), they have been shown to be slower than an algorithm that uses ideas from machine learning and tailored preconditioning (7). In this paper, we outline the process used to find the winning algorithm and share the insights obtained along the way. For context, the task had to be completed within the Synergistic Image Reconstruction Framework (SIRF) (8), and speed was measured as walltime until an application-focused convergence criterion was reached.

### 1.2 Problem details

Traditionally, fast algorithms for PET reconstruction have been subset-based (9), meaning only a subset of the data is used in every iteration. In the last decade, algorithms using a similar strategy but derived for machine learning have entered the field and shown state-of-the-art performance (7, 10–14). They exploit the fact that the KL divergence is separable in the estimated data

(2) $$D(Ax+r,y)=\sum_{i=1}^{n}\sum_{\;j\in S_{i}}d(A_{j}x+r_{j},y_{j}),$$

where n denotes the number of subsets and function d is defined by

$$d(s,t)=\left\{ \begin{array}{cc} s-t+t\log\;(t/s), & \text{if}\;t>0,s>0\\ s, & \text{if}\;t=0,s\geq 0\\ \infty , & \text{otherwise} \end{array}\right..$$

Here, $S_{i}$ denotes a subset of the data, e.g., all data associated to a “view.”

A great deal of effort has been put into finding good prior models (i.e., regularizers) for PET, including smooth and non-smooth priors, which either promote smoothness of the image to be reconstructed or encourage similarity to anatomical information (15–18). In Nuyts et al. (1), the authors proposed a smooth and convex prior that takes into account the scale of typical PET images, resulting in greater smoothness in less active regions. Mathematically, for non-negative images x, the resulting regularizer can be defined by

(3) $$S(x)=\frac{1}{2}\sum_{i}\sum_{\;j\in N_{i}}w_{i,j}\kappa_{i}\kappa_{j}\frac{{\left(x_{i}-x_{j}\right)}^{2}}{x_{i}+x_{j}+\gamma |x_{i}-x_{j}|+\varepsilon },$$

where the first sum is over all voxels i and the second sum is over all “neighbors” j. Parameter γ>0 allows placing more or less emphasis on edge preservation, and parameter ε>0 ensures that the function is well-defined and twice continuously differentiable. Terms $w_{i,j}$, $\kappa_{i}$, and $\kappa_{j}$ are weight factors accounting for distances between voxels and are intended to create a uniform “perturbation response” (19). Note that the essential part of the prior is

$$\phi (s,d)=\frac{d^{2}}{s+\gamma |d|+\varepsilon },$$

which has two important properties. First, if the sum of activities s between voxels is small compared to the scaled absolute difference γ|d|, the regularizer essentially reduces to total variation: ϕ(s,d)≈|d|/γ. Second, the larger the activity in both voxels, i.e., the larger s, the less weight is placed on penalizing their difference, justifying the name of the regularizer. See also Appendix A1 for formulas of derivatives.

Combined with the indicator function of the non-negativity constraint,

$$\iota_{\geq 0}(x)=\left\{ \begin{array}{cc} 0, & \text{if}\;x_{i}\geq 0\;\text{\textbackslash{},for all}\;i\\ \infty , & \text{otherwise} \end{array}\right.,$$

we arrive at the regularization model used in PETRIC

(4) $$R(x)=\beta S(x)+\iota_{\geq 0}(x).$$

This formula has to be interpreted to be ∞ for infeasible images with negative voxel values and has the finite RDP value everywhere else.

The rest of the paper is structured as follows. In Section 2, we introduce the building blocks of our algorithms and discuss proximal stochastic gradient approaches for solving Equation 1, stepsize regimes, preconditioning, and subset selection. In Section 3, we thoroughly investigate the effects of different choices of building blocks in a simulated setting. In Section 4, we present the algorithms we ended up using in PETRIC and their performance on real data. We conclude with final remarks in Sections 5 and 6.

## 2 Building blocks

Combining the modeling choices in Equations 1, 2, and 4, we arrive at the optimization problem

(5) $$\min_{x}\left\{\sum_{i=1}^{n}J_{i}(x)+\iota_{\geq 0}(x)\right\},$$

where we define $J_{i}(x)=D_{i}(x)+\frac{\beta }{n}S(x)$ and $D_{i}(x)\;:=\sum_{\;j\in S_{i}}d(A_{j}x+r_{j},y_{j})$. The variety of optimization methods for solving instances of problem 5 is extensive and has grown in recent decades; see Ehrhardt et al. (13) and references therein. For linear inverse problems, such as in PET image reconstruction, the most common approaches are based on either (proximal) gradient descent or on primal-dual approaches.

In this work, we consider stochastic gradient methods for solving problem 5. They take the form

(6) $$x^{\left(k+1\right)}={\;\mathrm{prox}}_{\iota_{\geq 0}}\left(x^{\left(k\right)}-\tau^{\left(k\right)}D^{\left(k\right)}{\tilde{\nabla }}^{\left(k\right)}\right),$$

where $\tau^{\left(k\right)}>0$ is a stepsize, ${\tilde{\nabla }}^{\left(k\right)}$ is an estimator of the gradient of the smooth part of the objective function $J(x)=\sum_{i=1}^{n}J_{i}(x)$, $D^{\left(k\right)}$ is a matrix that acts as a preconditioner (PC), and ${\;\mathrm{prox}}_{\iota_{\geq 0}}$ is the proximal operator associated with the non-negativity constraint, which can be efficiently computed entrywise, ${\left[{\;\mathrm{prox}}_{\iota_{\geq 0}}(x)\right]}_{j}=\max(0,x_{j})$.

All three components ${\tilde{\nabla }}^{\left(k\right)}$, $D^{\left(k\right)}$, and $\tau^{\left(k\right)}$ are critical for fast and stable algorithmic performance. In realistic image reconstruction settings, and in the context of the PETRIC challenge, the selection of these three components must balance accuracy and computational costs. In the remainder of this section, we review stochastic estimators, discuss their trade-offs, and address the stepsize selection and preconditioners. Finally, we consider the role of subset selection and sampling regimes, namely, how to choose the sets $S_{i}$ in Equation 2 and decide which subsets to use at each iteration of the algorithm.

### 2.1 Stochastic gradient methods

Let us turn our attention to the selection of gradient estimators ${\tilde{\nabla }}^{\left(k\right)}$.

**Stochastic gradient descent (SGD)** defines the gradient estimator by selecting a random subset index $i_{k}$ in each iteration and evaluating

$${\tilde{\nabla }}^{\left(k\right)}\;:=n\nabla J_{i_{k}}(x^{\left(k\right)})$$

to compute the update in Equation 6. Each iteration only requires storing the current iterate and computing the gradient for only one subset function. This can lead to large variances across updates, which increase with the number of subsets. To moderate this, vanishing stepsizes, satisfying

$$\sum_{k=1}^{\infty }\tau^{\left(k\right)}=\infty \;\text{and}\;\sum_{k=1}^{\infty }{\left(\tau^{\left(k\right)}\right)}^{2}<\infty ,$$

are required to ensure convergence but at the cost of convergence speed.

**Stochastic averaged gradient amelioré (SAGA)** controls the variance by maintaining a table of historical gradients ${\left(g_{i}^{\left(k\right)}\right)}_{i=1}^{n}\in X^{n}$. Each iteration uses a computed subset gradient combined with the full gradient table to update the gradient estimator

(7) $${\tilde{\nabla }}^{\left(k\right)}=n\left(\nabla J_{i_{k}}(x^{\left(k\right)})-g_{i_{k}}^{\left(k\right)}\right)+\sum_{i=1}^{n}g_{i}^{\left(k\right)},$$

followed by updating the corresponding entry in the table

$$g_{\;j}^{\left(k+1\right)}=\left\{ \begin{array}{cc} \nabla J_{i_{k}}(x^{\left(k\right)}), & \text{if}\;j=i_{k}\\ g_{\;j}^{\left(k\right)}, & \text{otherwise} \end{array}\right..$$

In contrast to SGD, SAGA guarantees convergence to a minimizer with constant stepsizes and preconditioners for Lipschitz-smooth problems. In its standard form, SAGA has the same computational cost as SGD, but it requires storing n gradients. The memory cost is not a practical limitation for most PET problems (even for relatively large n). If this is a concern, alternative formulations of SAGA exist with other memory footprints; see Ehrhardt et al. (13) for a further discussion.

**Stochastic variance reduced gradient (SVRG)** also reduces the variance by storing reference images and gradients, but unlike SAGA, these are updated infrequently. Algorithmically, SVRG is usually implemented with two loops: an outer loop and an inner loop. At the start of each outer loop, subset gradients and the full gradient estimator are computed at the last iterate as

$${\hat{g}}_{i}=\nabla J_{i}(\hat{x}),\;\hat{g}=\sum_{i=1}^{n}{\hat{g}}_{i}.$$

In the inner loop, the gradients are retrieved from memory and balanced against a randomly sampled subset gradient at the current iterate, giving the gradient estimator

(8) $${\tilde{\nabla }}^{\left(k\right)}=n(\nabla J_{i_{k}}(x^{\left(k\right)})-{\hat{g}}_{i_{k}})+\hat{g}.$$

Note the similarity between the gradient estimators of SAGA and SVRG given by Equations 7 and 8, respectively.

After ωn iterations, the snapshot image and the full gradient estimator are updated. The update parameter ω∈N is typically chosen as 2 for convex problems.

It is most common to store only the snapshot image $\hat{x}$ and the corresponding full gradient $\sum_{i=1}^{n}{\hat{g}}_{i}$, which then requires recomputing the subset gradient ${\hat{g}}_{i_{k}}$ at each iteration. This lowers the memory footprint (requiring only the snapshot image and the full gradient to be stored) but increases the computational costs.

### 2.2 Stepsizes

Theoretical convergence guarantees often require stepsizes based on $L_{\max}=\max_{i=1,\ldots ,n}\{L_{i}\}$, where $L_{i}$ is the Lipschitz constant of $\nabla J_{i}$. In PET, global Lipschitz constants are usually pessimistic, yielding conservative stepsize estimates.

Many stepsize approaches exist for stochastic iterative methods, ranging from predetermined choices made before running the algorithm (constant or vanishing) to adaptive methods [e.g., Barzilai–Borwein (BB) (20) and “difference of gradients”-type (21) rules] and backtracking techniques [e.g., Armijo (22)]. Due to the constraints imposed by the challenge (where computational time is a key metric), in this work, we focus on the first two categories.

**Constant** is the baseline stepsize rule. The specific value requires tuning to ensure convergence.

**Vanishing** rules consider stepsizes of the form $\tau^{\left(k\right)}=\tau^{\left(0\right)}/(1+\eta k/n)$, which satisfy the SGD convergence conditions, for $\tau^{\left(0\right)}>0$ and the decay parameter η>0 that needs to balance convergence and stability: small enough to maintain speed but large enough to ensure convergence.

**Adaptive** stepsize tuning via the BB rule is achieved by minimizing the residual of the secant equation at the current iterate. It converges for strongly convex problems and is applicable to SGD and SVRG (20). We tested several variants of the BB rule (long and short forms, geometric mean combinations, diagonal BB, etc.) but settled on the short-form BB for its performance and stability. When applied to gradient descent, short-form BB sets the stepsizes according to $\tau^{\left(k\right)}=p^{⊤}q/(q^{⊤}q)$, where $p=x^{\left(k\right)}-x^{\left(k-1\right)}$ and $q={\tilde{\nabla }}^{\left(k\right)}-{\tilde{\nabla }}^{\left(k-1\right)}$. When applied to SVRG, these values are computed during the iterations when the full gradient is recomputed.

### 2.3 Preconditioning

Preconditioners are essential for accelerating iterative reconstruction algorithms by stabilizing admissible stepsize and adapting them to individual components of the solution. Effectively, image components with large gradient variance receive smaller updates, and vice versa. This can have a dramatic effect in PET image reconstruction (and machine learning applications) due to the widely varying range of local Lipschitz constants. Motivated by Newton’s method, many preconditioners aim to approximate the inverse of the Hessian to allow for unit stepsizes. However, computing a full Hessian is impractical in high-dimensional problems, motivating the need for efficient approximations.

Preconditioners based only on data fidelity are standard in PET. The most prominent example is

$$D_{\mathrm{MLEM}}(x)=\mathrm{diag}\left(\frac{x+\delta }{A^{⊤}1}\right),$$

which can be derived from the gradient descent interpretation of MLEM. Here, the division of the two vectors is interpreted componentwise. Since x≥0 and $A^{⊤}1>0$, a small constant δ>0 ensures that the every diagonal entry of the preconditioner is non-zero. $D_{\mathrm{MLEM}}$ tends to work well for weak priors (e.g., in low-noise scenarios). However, it often underperforms because it does not account for the strength of the prior. This can either jeopardize the convergence behavior or require significant stepsize tuning.

Let

$$D_{\beta S}(x)=\mathrm{diag}\left(\frac{1}{\mathrm{diag}(H_{\beta S}(x))}\right)$$

be the inverse of the diagonal of the Hessian of the prior. In this work, we used diagonal preconditioners that combine the data fidelity and prior terms via the (scaled) harmonic mean between $D_{\mathrm{MLEM}}$ and $D_{\beta S}$. For scalars a,b>0, the harmonic mean is given by

$$h(a,b)=\frac{2}{\frac{1}{a}+\frac{1}{b}}.$$

Since our preconditioners are diagonal, this concept can be readily extended to define for some α>0

(9) $$\begin{array}{rl} D(x) & =\frac{1}{2}h(D_{\mathrm{MLEM}}(x),\alpha^{-1}D_{\beta S}(x))\\ & ={\left(D_{\mathrm{MLEM}}^{-1}(x)+\alpha D_{\beta S}^{-1}(x)\right)}^{-1}\\ & =\mathrm{diag}\left(\frac{x+\delta }{A^{⊤}1+\alpha \mathrm{diag}(H_{\beta S}(x))(x+\delta )}\right). \end{array}$$

Note that it satisfies $D(x)\leq \min\{D_{\mathrm{MLEM}}(x),\alpha^{-1}D_{\beta S}(x)\}$. While this may look like an *ad hoc* choice, if $D_{\mathrm{MLEM}}$ and $\alpha^{-1}D_{\beta S}$ are good approximations to their respective Hessians, then the harmonic mean D will be a good approximation to Hessian of the entire smooth term J. Note also that by the definition of the harmonic mean, the proposed preconditioner is diagonal with strictly positive diagonal elements. As such, standard results on convergence follow, e.g., with sufficiently small stepsizes.

We tested several alternatives to Equation 9, such as taking an componentwise minimum between $D_{\mathrm{MLEM}}$ and $D_{\beta S}$, reweighing their contributions, using the Kailath variant of the Woodbury identity (together with the diagonal approximation) to estimate the inverse of the Hessian, and other variants. The selected preconditioner provided the best balance between computational cost and algorithmic performance. Traditional second-order methods update the preconditioner in every iteration, which is costly. Preconditioner (Equation 9) is much cheaper and, as experiments show, requires updating only in the first three epochs, after which it stabilizes with no performance gain from further updates.

### 2.4 Subset selection and sampling

Subset-based reconstruction algorithms enhance the convergence speed of traditional iterative methods by dividing the projection data into multiple subsets and performing updates using partial measurement data. While this approach can offer significant computational advantages, careful selection of the number of subsets is critical. Using too many subsets can introduce artifacts and amplify noise, especially when subsets lack sufficient angular coverage, and increases the variance between successive updates, which can compromise the stability and convergence properties. Conversely, selecting too few subsets diminishes the acceleration benefit and causes behavior similar to classical methods, such as MLEM, which are known for their slow convergence. The number of subsets n is typically chosen as a divisor of the total number of projection angles (or views), allowing the data to be partitioned evenly. Subsets are then constructed to ensure that each is representative and uniformly distributed. We found that using approximately 25 subsets provides a good trade-off between reconstruction quality and computational speed in most scenarios, given the current computational requirements and scanner configurations.

To determine the order in which subsets are accessed, we consider the following standard choices:

**Herman–Meyer order (23)** is a well-established deterministic choice based on the prime decomposition of the number of subsets.

**Uniformly random with replacement** is the most common choice in machine learning applications. In each iteration, the subset index i is chosen by taking a sample from {1,…,n} uniformly at random.

**Uniformly random without replacement** randomizes access to subset indices but ensures that over n successive iteration cycles, all data are used by computing a permutation of (1,…,n) in each epoch.

**Importance sampling** uses a weighted variant of uniform sampling with replacement. For each 1≤i≤n, we assign a probability $p_{i}\geq 0$, such that $\sum_{i=1}^{n}p_{i}=1$. When Lipschitz constants $L_{i}$ are known, $p_{i}=L_{i}/\sum_{\;j=1}^{n}L_{j}$ is a common choice.

Since Lipschitz constants $L_{i}$ are unknown in PET, we propose an alternative importance sampling strategy for SVRG. Namely, when the full gradient estimator is updated, we compute $p_{i}=\|\nabla J_{i}(x)\|/\sum_{\;j=1}^{n}\|\nabla J_{j}(x)\|$, where x is the current image estimate. This incurs minimal computational overhead since all subset gradients are already recomputed in SVRG.

Finally, drawing inspiration from the Herman–Meyer ordering, which is designed to maximize information gain between successive updates and incorporating the concept of random sampling without replacement to ensure full coverage of subsets in each epoch with varying order, we propose the following novel subset ordering strategy.

**Cofactor order** begins by identifying all generators of the cyclic group associated with the number of subsets, n, which are identified as positive integers k<n that are coprime with n, meaning that they share no prime factors with it. These generators are then ranked according to their proximity to two reference points, 0.3n and 0.7n, to balance spread and randomness. In each epoch, the next available generator from this sorted list is selected and used to define a new traversal of the cyclic group, thereby determining the order in which subsets are accessed (i.e., one subset index per iteration). Once the list of generators has been exhausted, it is reinitialized, and the process repeats for subsequent epochs.

For example, if n=15, the coprimes (i.e., the set of generators) are given by {2,4,7,8,11,13,14}. The sorted list of coprimes, based on their proximity to 0.3n and 0.7n, is (4,11,2,8,7,13,14). Thus, 4 will be the first generator, which produces the subset indices: 0, 4, 8, 12, 1, 5, 9, 13, 2, 6, 10, 14, 3, 7, and 11. This exhausts the set of possible indices, so the next generator is selected as 11, and the process is repeated.

## 3 Numerical simulation experiments

To validate and refine the algorithmic components introduced in the previous section, we conducted a comprehensive suite of fast *inverse-crime* simulations. By simulating a simplified yet realistic PET scanner using the pure GPU mode of parallelproj v1.10.1 (24), iterative reconstructions could be run in seconds. This enabled a systematic exploration of the effects of various factors on convergence behavior, including the choice of stochastic algorithm, preconditioner, stepsize strategy, number of subsets, subset sampling method, time-of-flight (ToF) vs. non-ToF data, count levels, and regularization strength.

### 3.1 Simulation setup

All experiments used a simulated cylindrical (polygonal) scanner with a diameter of 600 mm and a length of 80 mm, comprising 17 rings with 36 modules each (12 detectors per module). Simulated ToF resolution was 390 ps, and a 4-mm isotropic Gaussian kernel in image space was used to model limited spatial resolution. Emission data were binned into a span 1 sinogram (289 planes, 216 views, 353 radial bins, 25 ToF bins). A simple 3D elliptical phantom was forward-projected (accounting for water attenuation), contaminated with a smooth background sinogram, and corrupted by Poisson noise to simulate realistic emission data. Low- and high-count regimes were simulated with $10^{7}$ and $10^{8}$ true events, respectively. Reconstruction was performed at an image size of 161×161×33 voxels with a 2.5 mm isotropic spacing.

**Figure 1:**
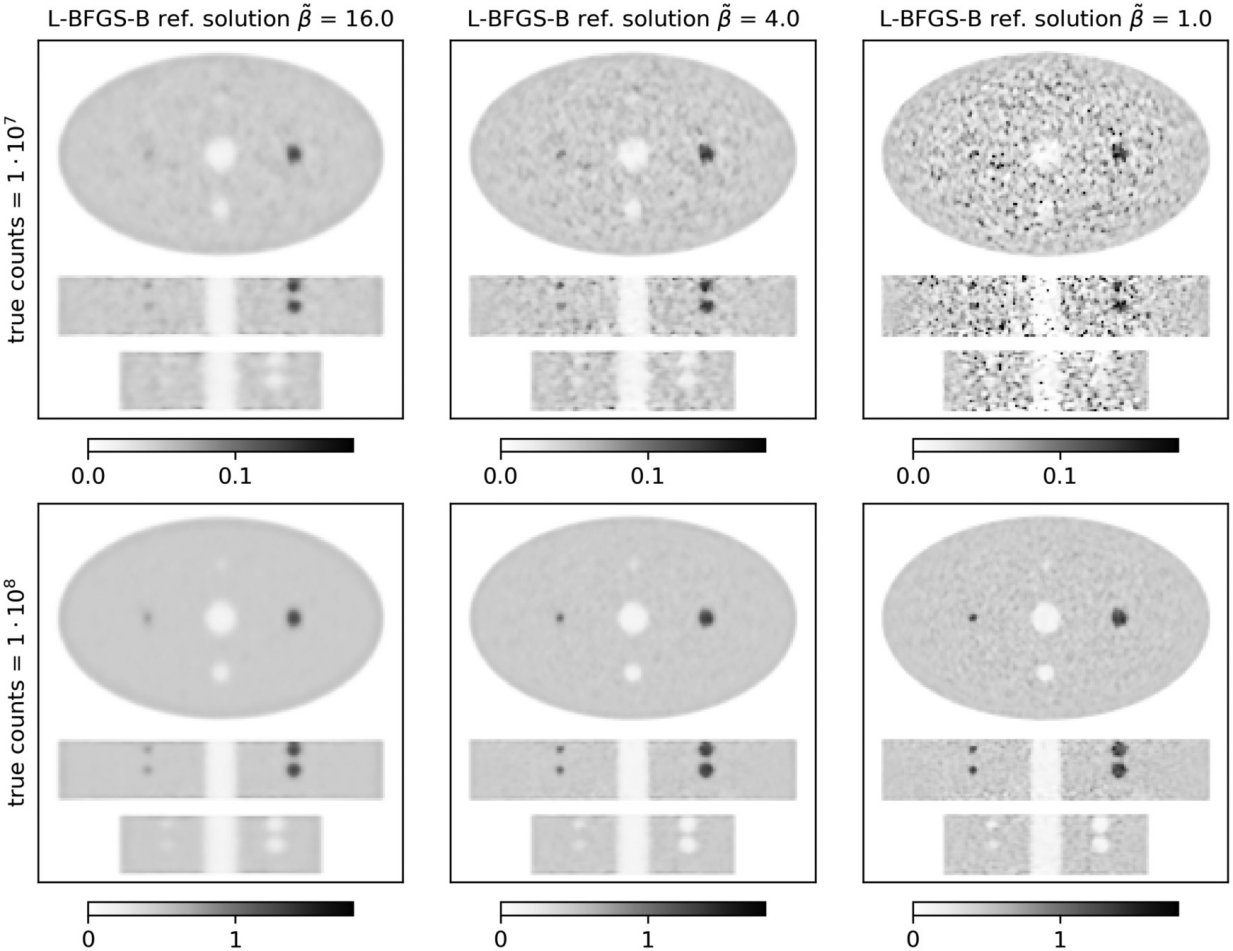
Stacked central transversal, coronal, and sagittal slices of L-BFGS-B reference reconstructions of the ellipse phantom. Each column shows a different level of regularization ($\tilde{\beta }$) increasing from left to right. The top row shows results for $10^{7}$ true counts, and the bottom row shows results for $10^{8}$ true counts.

Reference reconstructions (see Figure 1) were obtained by running 500 iterations of the preconditioned limited memory Broyden–Fletcher–Goldfarb–Shanno algorithm (L-BFGS-B) (25) with three relative regularization strengths $\tilde{\beta }\in \{1,4,16\}$. The regularization parameter β was scaled as

$$\beta =\tilde{\beta }\times 2\times 10^{-4}\times \frac{\text{true counts}}{3\times 10^{7}}.$$

This ensures that reconstructions with the same $\tilde{\beta }$ at different count levels show comparable resolution. All stochastic reconstructions were initialized with one epoch of ordered subset expectation maximization (OSEM) (with 27 subsets). Convergence was measured by the normalized root mean square error (NRMSE) excluding cold background around the elliptical phantom, normalized by the intensity of the largest background ellipsoid. In line with the NRMSE target threshold used in the PETRIC challenge, we consider the point where NRMSE was less than 0.01 as a marker of practical convergence. The data were divided into n subsets by selecting every nth view. Unless stated otherwise, in each epoch, subsets were drawn uniformly at random without replacement. All runs were performed using an NVIDIA RTX A4500 GPU. The code for all our simulation experiments and submissions to PETRIC is available at https://github.com/SyneRBI/PETRIC-MaGeZ. To reproduce the results, users should use the tagged versions ALG1, ALG2, ALG3, or 2024_paper_simulation_results.

### 3.2 Main simulation results

**Figure 2:**
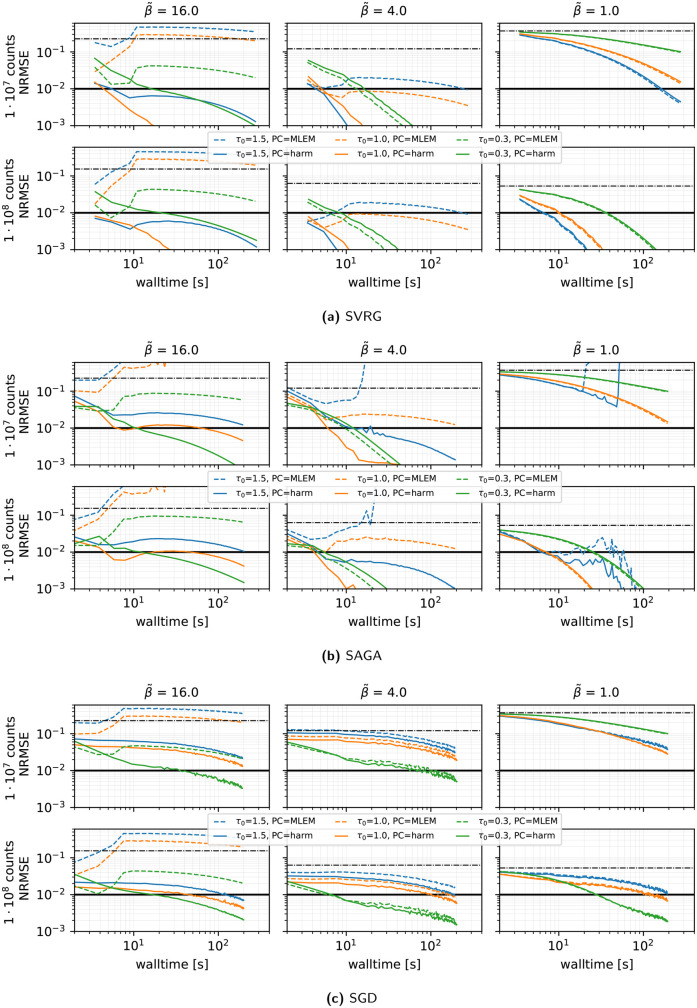
Reconstruction performance in terms of NRMSE vs. walltime for **SVRG**, **SAGA**, and **SGD**, for MLEM (dashed lines) and harmonic (solid lines) PCs and three initial stepsizes ($\tau^{\left(0\right)}$) represented by different colors, using 27 subsets, a gentle stepsize decay with η=0.02,100 epochs, and subset selection without replacement. Results are shown for three levels of regularization ($\tilde{\beta }$) and two count levels. Note the logarithmic scale on the *x*- and *y*-axes. For each combination of preconditioner and $\tau^{\left(0\right)}$, the outcome of **one run** is displayed. The thick solid line shows the NRMSE target threshold of $10^{-2}$ used in the PETRIC challenge, and the dashed–dotted horizontal black line shows the NRMSE of the initial OSEM reconstruction.

**Algorithm and preconditioner effects (see Figure 2):** When comparing SVRG, SAGA, and plain SGD under a vanishing stepsize schedule $\tau^{\left(k\right)}=\tau^{\left(0\right)}/(1+0.02\;k/n)$ with $\tau^{\left(0\right)}\in \{0.3,1.0,1.5\}$ and n=27, we made the following observations.

• SVRG and SAGA consistently outperform SGD in all count and regularization regimes.
• The harmonic mean preconditioner (Equation 9) is crucial: under strong regularization $\tilde{\beta }=16$, the classic MLEM preconditioner diverges or converges extremely slowly (depending on the chosen stepsize), whereas the harmonic mean variant converges reliably in every scenario.
• SVRG with the harmonic preconditioner, $\tau^{\left(0\right)}=1$ and η=0.02 (giving mild decay), yields the fastest convergence for medium and high $\tilde{\beta }$. For low regularization, a slightly larger $\tau^{\left(0\right)}$ (up to 1.5 or 2.5) can accelerate convergence.
• Across all methods, convergence was slower in the case of low regularization $\tilde{\beta }=1$.

**Figure 3:**
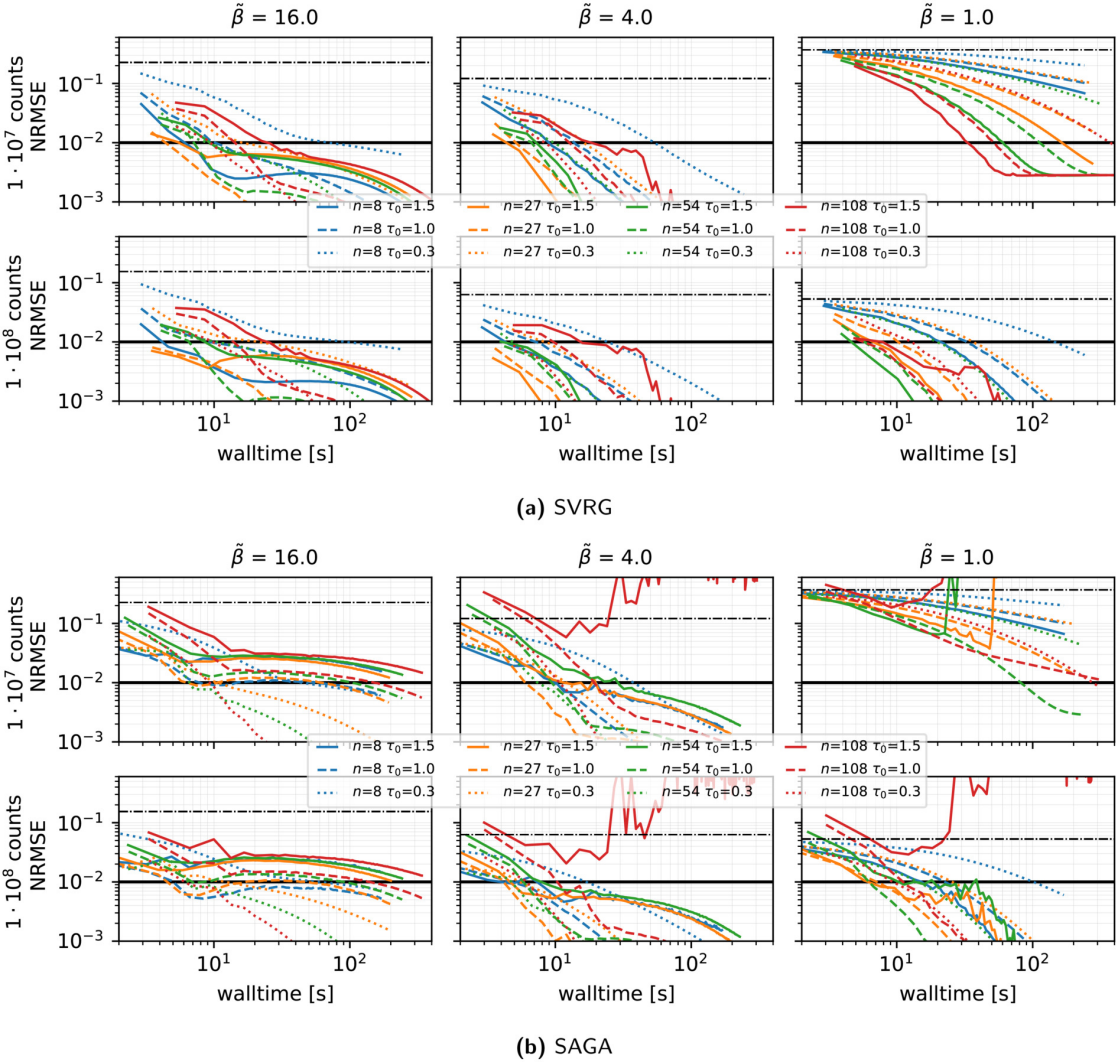
Performance in terms of NRMSE vs. walltime for **SVRG** and **SAGA**, for different number of subsets n and initial stepsizes $\tau^{\left(0\right)}$, using the harmonic preconditioner, a gentle stepsize decay with η=0.02,100 epochs, and subset selection without replacement. Results are shown for three levels of regularization $\tilde{\beta }$ and two count levels. For each combination of n and $\tau^{\left(0\right)}$, the outcome of **one run** is displayed. The thick horizontal black line shows the NRMSE target threshold of $10^{-2}$ used in the PETRIC challenge.

**Impact of the number of subsets (see Figure 3):** Fixing the harmonic preconditioner and vanishing stepsize rule $\tau^{\left(0\right)}=1,\eta =0.02$, we varied the number of subsets n∈{8,27,54,108}:

• SVRG achieves optimal walltime convergence at n=27 under medium to high $\tilde{\beta }$. Lower $\tilde{\beta }$ benefits from using a greater number of subsets.
• Optimal values of n and $\tau^{\left(0\right)}$ for SAGA depend strongly on $\tilde{\beta }$: high $\tilde{\beta }$ favors a larger number of subsets with smaller $\tau^{\left(0\right)}$, medium $\tilde{\beta }$ favors n=27 with $\tau^{\left(0\right)}\approx 1$, and low $\tilde{\beta }$ favors n≈54.
• Overall, SVRG with optimized settings achieves faster convergence compared to SAGA with optimized settings.

**Figure 4:**
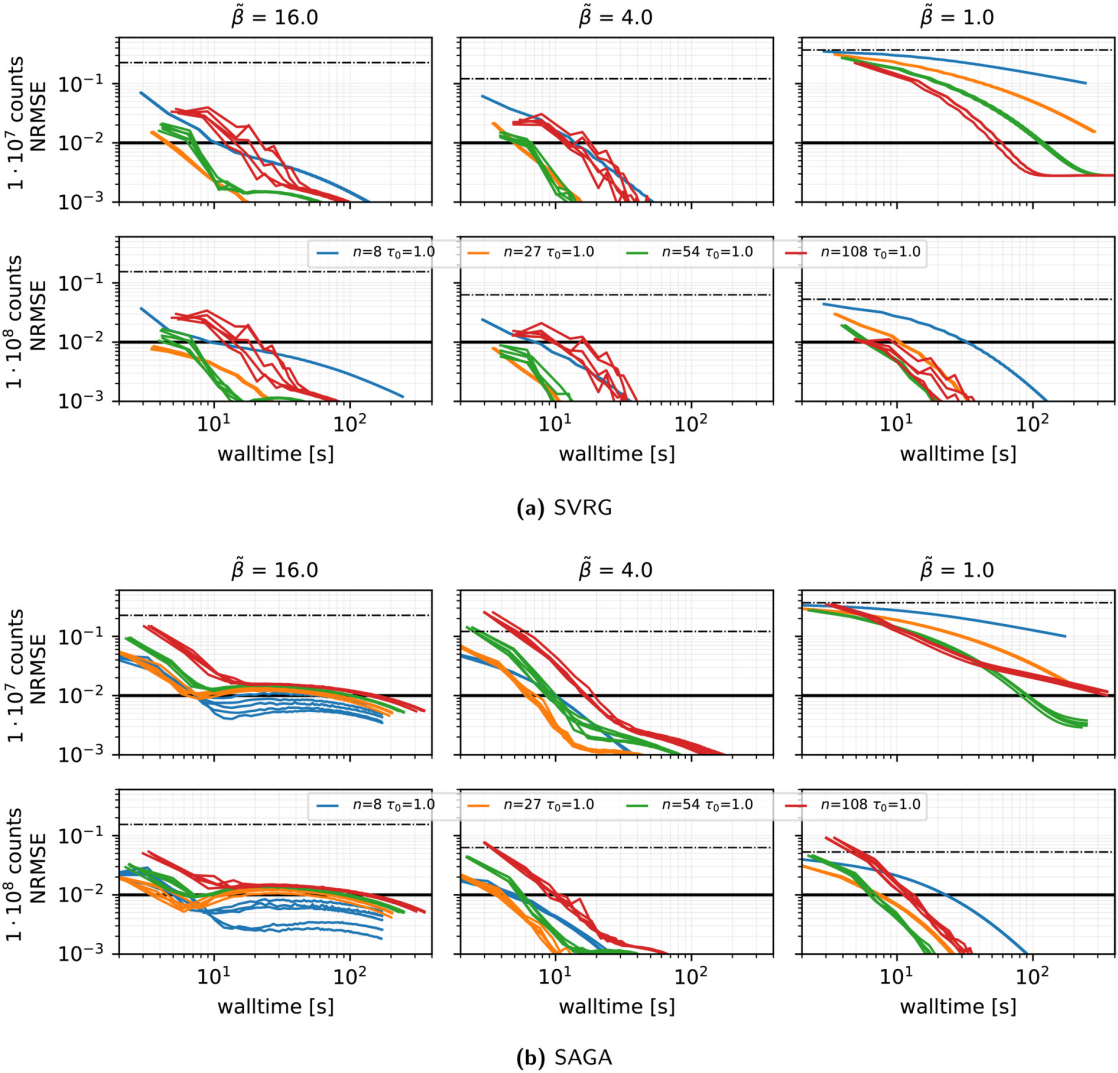
Same as Figure 3 showing the results of **five runs**, using a different random seed for the subset selection.

**Stability across repeated runs using different subsets orders (see Figure 4):** We run five independent runs (changing the random seed used for the random subset selection) of the reconstructions using SVRG, the harmonic preconditioner, $\tau^{\left(0\right)}=1$, η=0.02, and n∈{8,27,54,108}. The run-to-run NRMSE variation is small, especially at n=27, confirming low variance introduced by the stochastic subset selection in this setting.

**Figure 5:**
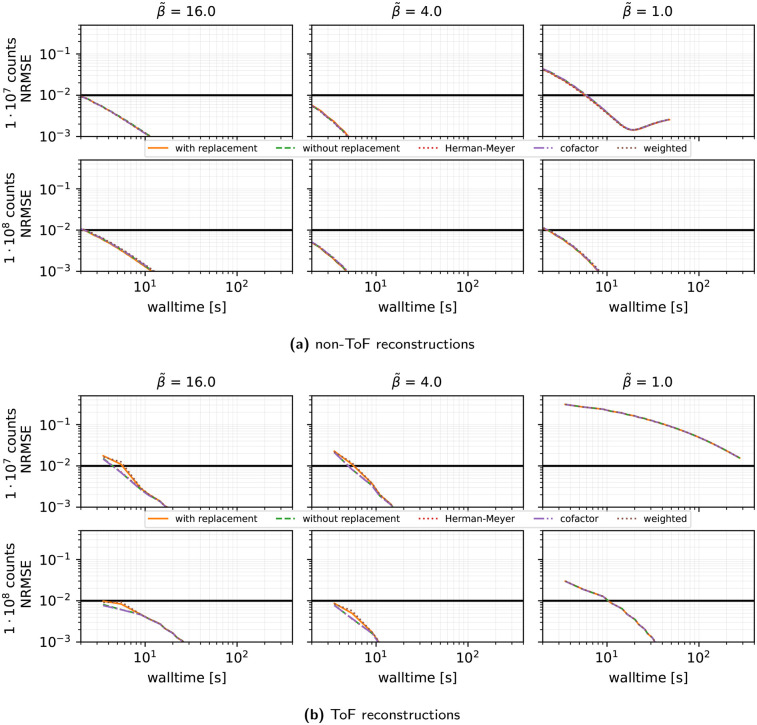
Same as Figure 3 (SVRG only) showing the results for different subset sampling strategies, n=27 subsets, the harmonic preconditioner, an initial stepsize $\tau^{\left(0\right)}=1$, and gentle stepsize decay using η=0.02 for non-ToF (top) and ToF reconstructions (bottom).

**Subset sampling strategy (see Figure 5):** Comparing the Herman–Meyer order, uniform sampling at random with and without replacement, importance sampling, and cofactor strategies for selecting the order of subsets for SVRG with $\tau^{\left(0\right)}=1$, n=27, and η=0.02, we observe negligible differences between all subset selection rules in simulated scenarios, with some minor benefits for sampling without replacement and cofactor sampling.

**Figure 6:**
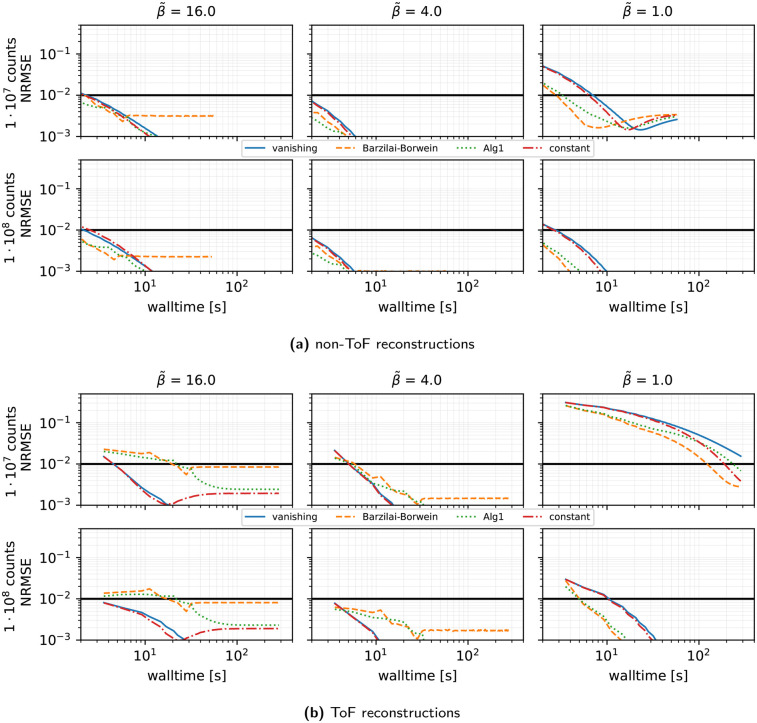
Same as Figure 5 showing the results for different stepsize strategies, 27 subsets, and the harmonic preconditioner for non-ToF (top) and ToF reconstructions (bottom).

**Stepsize rules (see Figure 6):** We see that for SVRG, n=27, and the harmonic preconditioner:

• At low $\tilde{\beta }$, adaptive rules (short-form BB or heuristic ALG1) modestly outperform a simple decay.
• However, in the medium-to-high $\tilde{\beta }$ regime, a constant or decaying initialization $\tau^{\left(0\right)}=1$ yields superior ToF reconstruction performance compared to adaptive BB schemes.

### 3.3 Simulation-derived conclusions

The inverse-crime simulation study motivated the design of our algorithms submitted to the PETRIC challenge in the following way:

• The **harmonic mean preconditioner** was essential for achieving stable convergence with $\tau^{\left(0\right)}\approx 1$ across different count and regularization regimes.
• **SVRG** slightly outperformed SAGA in robustness and speed, and both outperformed SGD.
• A moderate number of subsets, n≈27, led to the fastest convergence times.

These guidelines directly informed our implementation choices for the three submitted algorithms, which are explained in detail in the next section.

## 4 Submitted algorithms and their performance

Based on the insights gained from the inverse-crime simulations in the previous section, we implemented and submitted three closely related algorithms (termed **ALG1**, **ALG2**, and **ALG3**) to the PETRIC challenge under the team name MaGeZ. All three algorithms use SVRG as the underlying stochastic gradient algorithm and apply the harmonic mean preconditioner (Equation 9). The pseudocode that forms the basis of all three algorithms is given in Algorithm 1 in Appendix A2. Our SVRG implementation uses in-memory snapshot gradients, adding only a small overhead compared to plain SGD or BRSEM. In the context of sinogram-based PET reconstructions of data from modern scanners, where gigabytes are devoted to storing sinograms, this extra memory requirement can be effectively neglected, as discussed in Twyman et al. (7).

The available PETRIC training datasets were primarily used to fine-tune the algorithm hyperparameters, namely, (i) the number of subsets, (ii) the subset selection strategy, (iii) the stepsize rule, and (iv) the update frequency of the preconditioner. These are the only distinguishing features among the submitted algorithms, and our choices are summarized in Table 1. ALG1 and ALG2 use the number of subsets as the divisor of the number of views closest to 25. ALG3 further modifies the subset count slightly using the divisor closest to 24.2 (with the goal of selecting a smaller number of subsets in some of the training datasets). In ALG1 and ALG2, subsets are chosen uniformly at random without replacement in each iteration of each epoch. ALG3 uses the proposed cofactor rule. ALG1 updates the preconditioner at the start of epochs 1, 2, and 3. ALG2 and ALG3 update the preconditioner at the start of epochs 1, 2, 4, and 6. ALG1 uses a fixed, piecewise stepsize schedule, while ALG2 and ALG3 employ a short BB rule for adaptive stepsize reduction, which is computed at the start of epochs 1, 2, 4, and 6.

**Table 1:** Key hyperparameters of the three submitted algorithms.

Property	ALG1	ALG2	ALG3
Gradient estimator	SVRG	Same as ALG1	Same as ALG1
Preconditioner	Harmonic mean	Same as ALG1	Same as ALG1
Preconditioner update epochs	1, 2, 3	1, 2, 4, 6	1, 2, 4, 6
Number of subsets	Divisor of the number of views closest to 25	Same as ALG1	Divisor of the number of views closest to 24.2
Subset selection rule	Fixed random sequence without replacement	Same as ALG1	Cofactor
Stepsize rule	$\left\{ \begin{array}{cc} 3 & k<10\\ 2 & 10\leq k<100\\ 1.5 & 100\leq k<200\\ 1 & 200\leq k<300\\ 0.5 & 300\leq k \end{array}\right.$	$\left\{ \begin{array}{cc} \min({\tau^{\left(k\right)}}_{\mathrm{bb}},3) & k<10\\ \min({\tau^{\left(k\right)}}_{\mathrm{bb}},2.2) & 10\leq k<2n\\ \min({\tau^{\left(k\right)}}_{\mathrm{bb}},1) & 2n\leq k \end{array}\right.$ with ${\tau^{\left(k\right)}}_{\mathrm{bb}}$, the short BB step, calculated at the end of epochs 2, 4, and 6.	Same as ALG2

### 4.1 Performance on PETRIC test datasets

**Figure 7:**
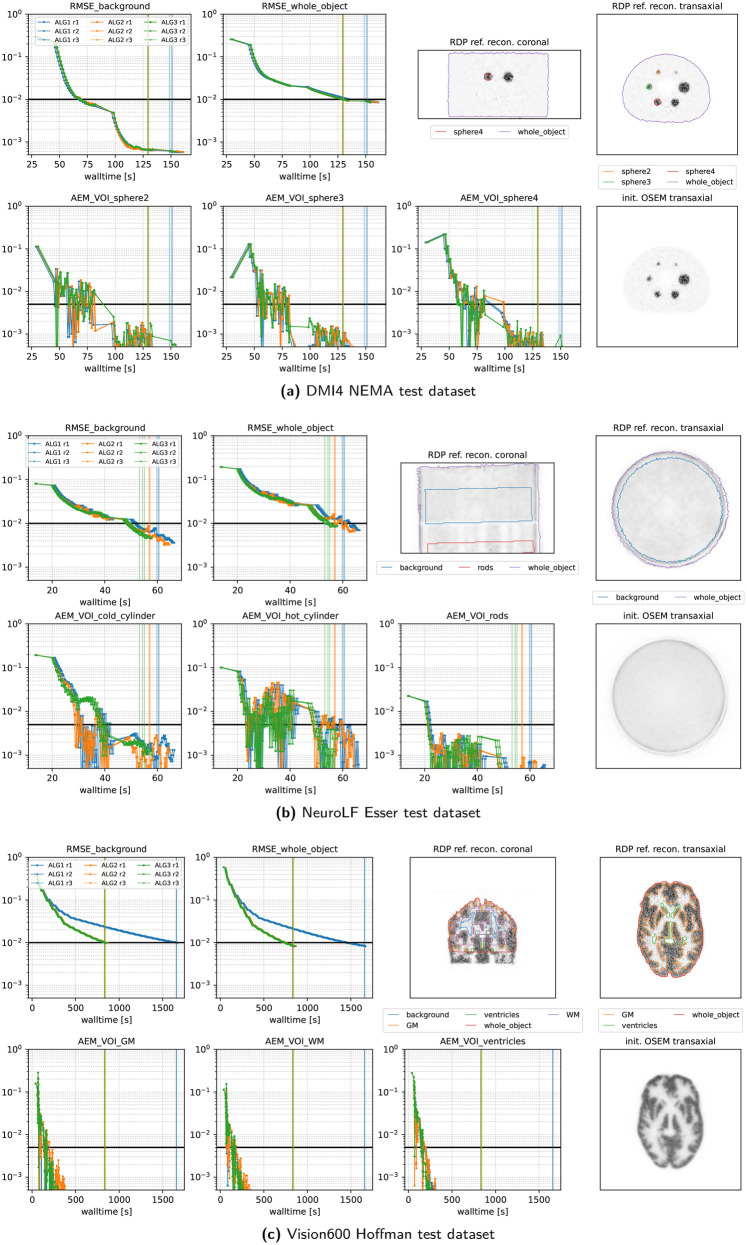
Performance metrics of our three submitted algorithms evaluated on three representative PETRIC test datasets using three repeated runs. The vertical lines indicate the time when the thresholds of all metrics were reached. Note the logarithmic scale on the *y*-axis and the linear scale on the *x*-axis. The top right images show coronal and transaxial slices of the reference reconstruction alongside contour lines of the volumes of interest used for the metrics. The bottom right image shows the same transaxial slice of the OSEM reconstruction used for the initialization of all algorithms.

**Figure 8:**
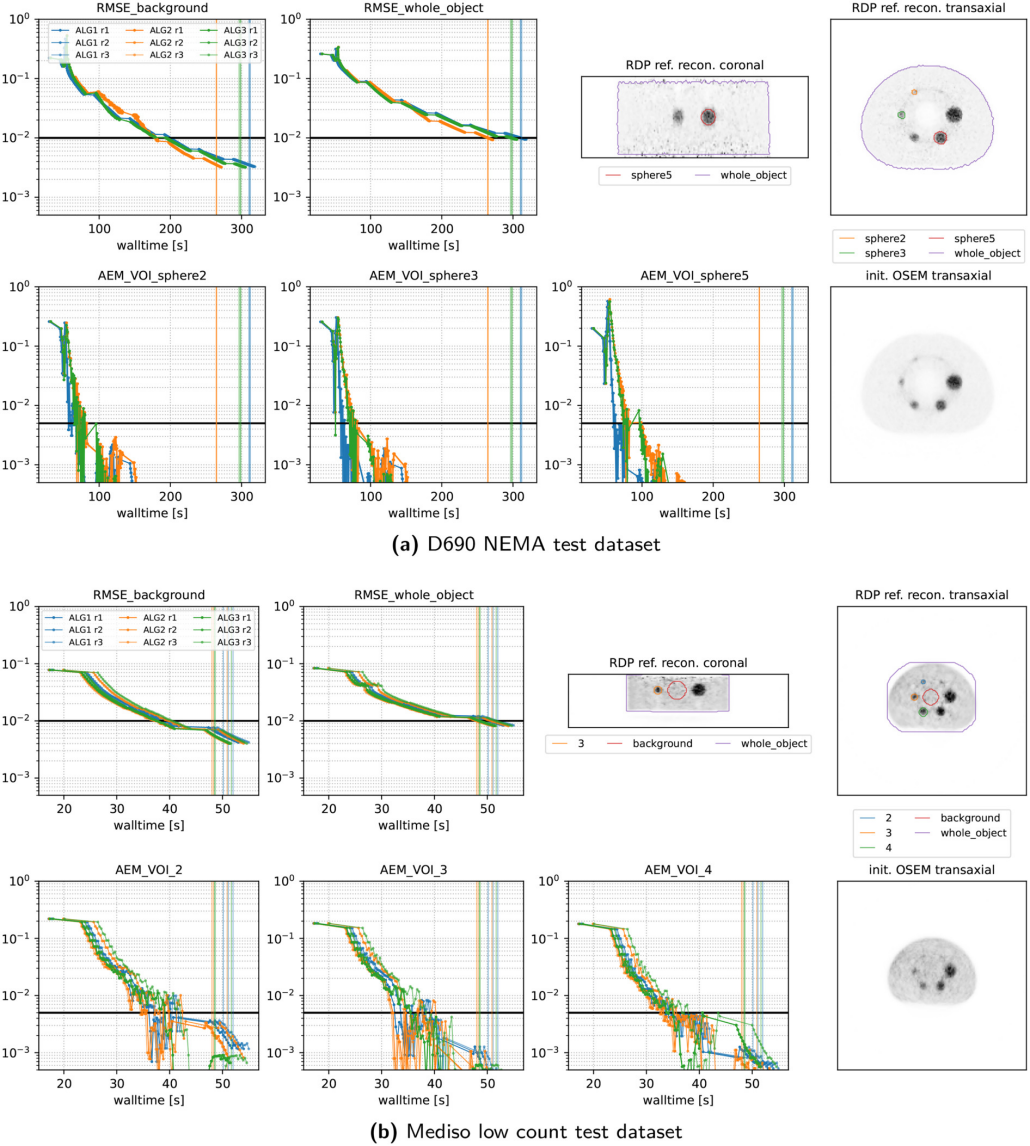
Same as Figure 7 for two more datasets.

Figures 7 and 8 present the convergence behavior of all three submitted algorithms in terms of whole-object NRMSE, background NRMSE, and multiple volume-of-interest (VOI) mean absolute error metrics (AEMs). Each dataset was reconstructed three times with all three algorithms using a local NVIDIA RTX A4500 GPU. From the two figures, we observe the following:

• **All algorithms converge** reliably across all datasets and runs.
• **ALG2 and ALG3 perform similarly** and slightly outperform ALG1 in most cases. In the Vision600 Hoffman dataset, ALG1 takes almost twice as long as ALG2 and ALG3 to reach the convergence threshold.
• **For the DMI4 NEMA, NeuroLF Esser, and Mediso low-count datasets**, convergence is reached very quickly both in terms of walltime and epoch count, typically within four epochs.
• **The Vision600 Hoffman dataset** shows the slowest convergence, requiring more than 23 epochs (594 updates) for ALG2 and ALG3 and more than 47 epochs (1,184 updates) for ALG1.
• **Inter-run variability** is low, with timing differences between runs being within 1–2 s.
• Across all datasets, **whole-object NRMSE is the slowest metric to converge**, becoming the bottleneck in determining the final convergence time.

A closer inspection of the stepsize behavior on the Vision600 Hoffman dataset reveals that the slower convergence of ALG1 is due to its lower final stepsize, which was implemented as a “safety feature.” After 300 updates, ALG1 reduces its $\tau^{\left(k\right)}$ to 0.5, whereas ALG2 and ALG3 continue to use $\tau^{\left(k\right)}=1.0$ since their BB-based calculated adaptive stepsizes remained larger in this dataset. This difference explains the kink observed in ALG1’s convergence curves around 450 s.

## 5 Discussion

We now want to discuss what we believe are the important and interesting aspects of this work.

In our view, the most important feature of our algorithms is the improved preconditioner, which takes into account Hessian information of the regularizer. This enhancement allowed for a better generalization of stepsize choices across a range of scanners, objects, noise levels, and regularization strengths. We chose SVRG as our gradient estimator, although this choice is not as clear-cut and might be different for other variants of the reconstruction problem. Our experience suggests that while a sophisticated method to control variance is important, the specific approach (e.g., SVRG or SAGA) appears to be less critical. In contrast, other factors like stepsizes and sampling strategies had a relatively minor impact, as the algorithms were not particularly sensitive to these choices.

A key aspect in our approach was to consider what could be effectively computed and what could not. For the RDP, it is easy to compute the gradient and the diagonal Hessian, but other operations such as the proximity operator or the full Hessian are much more costly. Similarly, the ideal number of subsets is largely a computational efficiency question. It has been observed numerous times that, theoretically, fewer epochs are needed with a larger number of subsets. However, practically, this means that the overhead per epoch increases, e.g., as the gradient is computed in each iteration of the epoch. These two factors must be traded off against each other.

Speaking of the RDP, we noticed a couple of interesting features that we have not exploited in our work. First, the diagonal Hessian of the RDP is very large in background regions where the activity is small. Second, while its gradient has a Lipschitz constant, similar to the total variation and its smoothed variants, algorithms that do not rely on gradients might be beneficial.

Between the three algorithms, ALG2 and ALG3 consistently performed either similarly to or better than ALG1. Comparing them to the submissions of other teams, it is worth noting that for almost all datasets, they performed far better than any of the other competitors, which lead to MaGeZ winning the challenge overall (26).

Coordination between our simulation insights and algorithm design was essential to our approach. Local testing allowed us to validate the generalization of our methods before final submission. Across datasets, we favored robustness over aggressive tuning. Refinement came from iterative testing rather than from theoretical guarantees alone. Above all, our goal was to develop an algorithm that performs well out-of-the-box.

## 6 Conclusions

In this paper, we presented our strategy and thought process behind designing our winning strategy for the 2024 PETRIC challenge. We identified the key parameters for PET image reconstruction algorithms using realistic yet very fast simulations. The harmonic mean preconditioner helped us to overcome the biggest roadblock of the challenge: tuning of parameters for a variety of settings with various scanner models, phantoms, and regularization strengths.

## Data Availability

The original contributions presented in the study are included in the article/Supplementary Material.

## Appendix

### 1 Gradient and Hessian of the RDP

For completeness, we present here the first and second derivatives of the RDP (Equation 3), i.e., the gradient and the diagonal of the Hessian. Both of these are used in our proposed solution.

Let $d_{i,j}=x_{i}-x_{j}$, $s_{i,j}=x_{i}+x_{j}$, and $\phi_{i,j}=s_{i,j}+\gamma |d_{i,j}|+\varepsilon$. Then the first derivative is given by

$$\partial_{x_{i}}S(x)=\sum_{\;j\in N_{i}}w_{i,j}\kappa_{i}\kappa_{j}\frac{d_{i,j}(2\phi_{i,j}-(d_{i,j}+\gamma |d_{i,j}|))}{\phi_{i,j}^{2}},$$

and the second by

$$\partial_{x_{i}}^{2}S(x)=2\sum_{\;j\in N_{i}}w_{i,j}\kappa_{i}\kappa_{j}\frac{{\left(s_{i,j}-d_{i,j}+\varepsilon \right)}^{2}}{\phi_{i,j}^{3}}.$$

### 2 Pseudocode for submitted preconditioned SVRG algorithm

Algorithm 1 Preconditioned SVRG algorithm.

**Require:** initial image: x, number of subsets: n, stepsize rule: stepsize, sampling rule: subset, diagonal preconditioner rule: preconditioner, list of iterations to update the preconditioner: update_pc_iters, update gradient at anchor point every ω epochs (default = 2)
1: **for** k=0,1,… **do**
2:  **if** k∈update_pc_iters **then**
3:   D←preconditioner(x) ▹ update preconditioner via Equation 9
4:  **end if**
5:  **if** kmod(ωn)=0 **then**
6:   **for** i=1 **to** n **do**
7:    ${\hat{g}}_{i}\leftarrow \nabla J_{i}(x)$ ▹ calculate all subset gradients at snapshot image
8:   **end for**
9:   $\hat{g}\leftarrow \sum_{i=1}^{n}{\hat{g}}_{i}$
10:   $\tilde{\nabla }\leftarrow \hat{g}$
11:  **else**
12:   i←subset(k)
13:   $\tilde{\nabla }\leftarrow n\left(\nabla J_{i}(x)-{\hat{g}}_{i}\right)+\hat{g}$
14:  **end if**
15:  τ←stepsize(k)
16:  $x\leftarrow x-\tau D\tilde{\nabla }$
17:  **if** stopping criterion is reached **then return** x
18:  **end if**
19: **end for**

**Table:**

BB	Barzilai–Borwein
KL	Kullback–Leibler
MLEM	Maximum likelihood expectation maximization
NRMSE	Normalized root mean square error
OSEM	Ordered subsets expectation maximization
PET	Positron emission tomography
PETRIC	PET rapid image reconstruction challenge
RDP	Relative difference prior
SAGA	Stochastic averaged gradient amelioré
SGD	Stochastic gradient descent
SIRF	Synergistic image reconstruction framework
SVRG	Stochastic variance reduced gradient
ToF	Time-of-flight
